# Mechanisms of natural killer cell-mediated clearance of senescent renal tubular epithelial cells

**DOI:** 10.3389/fcell.2025.1597230

**Published:** 2025-06-30

**Authors:** Nils David Funk, Julius Sinning, Patrick Schaser, Inga Soerensen-Zender, Vera Christine Wulfmeyer, Kai Schmidt-Ott, Korbinian Brand, Stephan Halle, Roland Schmitt

**Affiliations:** ^1^ Department of Nephrology and Hypertension, Hannover Medical School, Hannover, Germany; ^2^ Institute of Clinical Chemistry and Laboratory Medicine, Hannover Medical School, Hannover, Germany; ^3^ Institute of Immunology, Hannover Medical School, Hannover, Germany; ^4^ Department of Nephrology and Hypertension, University Hospital Schleswig-Holstein, Kiel, Germany

**Keywords:** senescence, NK cells, kidney, PTEC, CKD - chronic kidney disease, immunosurveillance, immune system

## Abstract

**Introduction:**

Intact kidney function is essential for fluid and electrolyte homeostasis, with renal tubular epithelial cells providing critical functions in the reabsorption or secretion of numerous metabolites. Cellular senescence of the renal epithelial cells can lead to functional deficits. Therefore, immune-cell-mediated clearance of senescent epithelial cells can be an important factor for the maintenance of kidney function.

**Methods:**

In this study, we established a model to directly monitor natural killer (NK) cell-mediated clearance of senescent renal tubular cells. To this end we used primary cell co-cultures with different read-outs and life cell imaging.

**Results and Discussion:**

We observed that the clustering of NK cells on senescent renal epithelial cells could be used to detect senescence-triggered NK cell activation. Also, we found that NKG2D signaling and perforin-dependent lysis of senescent renal epithelial cells were crucial steps in the lysis of senescent cells. In addition, NK cell-mediated attack of senescent renal epithelial cells could be dampened by the addition of cyclosporine A and was augmented by the addition of interleukin 7. Together, these data show that NK cells can efficiently mediate the clearance of senescent renal tubular epithelial cells involving NK cell activation by NKG2D signaling.

## Introduction

Chronic kidney disease (CKD) affects a rapidly growing population worldwide. Patients with CKD are at a strongly increased risk for overall morbidity and mortality, and it is projected that by the year 2040, CKD will be the fifth leading cause of years of life lost in the world (Foreman et al., 2018). Advanced age is the strongest single risk factor for CKD and due to several overlapping features with physiological kidney aging CKD is often regarded as an accelerated aging process (Rex et al., 2023). One of the typical biological changes, which can be observed in both CKD and the old kidney, is an accumulation of cellular senescence (Mylonas and Ferenbach, 2024).

Cellular senescence is an irreversible cell cycle arrest, which is associated with changes in chromatin organization, gene transcription, metabolism and protein secretion. While cellular senescence is a crucial physiological process during healthy development, it can be harmful during later life if senescent cells accumulate in the body (Gorgoulis et al., 2019). Generally, cellular senescence occurs as a response to cellular stress, such as telomere shortening, DNA damage, oncogene activation, hypoxia, toxin exposure, energy or nutrient deprivation during aging and disease states (Muñoz-Espín and Serrano, 2014). Senescent cells upregulate different cell cycle inhibitors, mainly p16INKA and/or p21cip1, and activate pro-survival pathways to resist apoptosis. In most cell types, the senescent state is associated with an increased activity of the lysosomal hydrolase senescence-associated β-galactosidase (SA-β-Gal) and with a distinctive secretory phenotype consisting of various pro-inflammatory molecules and growth factors, known as senescence-associated secretory phenotype (SASP) (Huang et al., 2022; Rex et al., 2023). SASP molecules are thought to be crucial for the pathophysiological effects of senescent cells as they elicit inflammatory processes in the surrounding tissue, which, in the absence of an adequate immune response, may progress to chronic inflammation, fibrosis and tissue dysfunction.

The detrimental role of cellular senescence in the kidney has been highlighted by experimental work, in which elimination of senescent cells improved kidney regeneration and antagonized the progression of CKD and age-related changes (Mylonas and Ferenbach, 2024). Several groups, including ours, have found this causative interrelationship in mouse models of acute kidney injury (AKI), CKD and kidney transplantation (Johmura et al., 2021; Mylonas et al., 2021; Li et al., 2023; Liao et al., 2022). While most of these studies achieved a clearance of senescent cells via the administration of cell death-inducing chemical agents, often referred to as “senolytics,” another strategy could involve immunotherapies, which foster the natural immune clearance of senescent cells (Mylonas and Ferenbach, 2024).

Physiologically, the immune system plays a crucial role in senescence surveillance by maintaining an ongoing clearance of senescent cells, with macrophages, T-cells and natural killer (NK) cells being the primary actors in this process (Kale et al., 2020). Through several still underexplored receptor-ligand interactions, immune cells are able to detect and eliminate senescent cells (Salminen, 2021). SASP factors are believed to shape the senescent microenvironment and set the ground for immune cell recruitment, but research on the involved signaling pathways has been challenging as the SASP composition is highly variable according to cell-type, senescence stage and senescent phenotype (Gorgoulis et al., 2019; Oguma et al., 2023). Importantly, for various reasons, immune cells may encounter challenges in an effective elimination mechanism. While immunological senescence surveillance is highly efficient during development, it is compromised in advanced age or in situations where the immune system is suppressed (e.g., by immunosuppressive drugs after allogenic organ transplantation). In these scenarios, senescent cells can accumulate in tissues, such as the kidney, and contribute to structural and functional decline (Moiseeva et al., 2023). Restoring the immune response towards the elimination of senescent cells may hold important therapeutic potential for improved outcomes in AKI, CKD and kidney transplantation (Kale et al., 2020; Matsunaga et al., 2021). As NK cells have been primarily involved in the elimination of senescent cells, adoptive NK cell therapy has been suggested as a strategy to fight age-related disease and promote longevity (Deng and Terunuma, 2024).

The interaction between immune cells and senescent cells of the kidney has not been studied in detail so far. In particular, there is a complete research gap about the relationship between NK cells and senescent tubular epithelial cells, the most abundant cell type of the kidney. A better understanding of this interaction constitutes a promising axis with potential opportunities for therapeutic strategies. Our primary goal in this study was therefore to characterize the interactions between NK cells and kidney tubular cells. To this end, we established a primary tubular epithelial cell (PTEC) *in vitro* model based on NK cell activity to identify decisive receptors and ligands involved in the cell-cell interaction with senescent target cells. Subsequently, we addressed the impact of exogenous substances and medications, which might modulate the mechanisms underlying the clearance of senescent tubular cells.

## Materials and methods

### Ethics statement

All mouse experimental protocols were approved by the local animal welfare authorities (Niedersaechsisches Landesamt für Verbraucherschutz und Lebensmittelsicherheit, LAVES, Oldenburg, Germany) and performed according to local animal welfare law (animal welfare protection act) under license number: 2022/306 (reviewed by LAVES).

### Extraction of murine primary cells

All mice were bred at Hannover Medical School (Central Animal Laboratory and Institute for Laboratory Animal Science) and housed under standard pathogen-free conditions in accordance with local animal welfare regulations. Primary tubular epithelial cells (PTEC) were isolated as described previously (Liao et al., 2022). PTEC were isolated from euthanized donor C57BL/6, B6N; 129-Ncr1tm1Oman/J, (B6 Ncr1GFPxB6, tm/wt), B6.129(ICR)Tg(CAGECFP)CK6Nagy/J or B6N; 129-NCr1tm1Oman/J (B6 GFP tm/tm) mice. Perforin-deficient NK cells were isolated from perforin-deficient C57BL/6-Prf1tm1Sdz/J mice (JAX stock #002407) as described previously (Halle et al., 2016; Lueder et al., 2018). Donor mice were euthanized by CO2 narcosis and cervical dislocation and kidneys were manually dissected and digested in 0.125% Collagenase Type I (Affymetrix/USB) solution (37°C; 45 min). The sediment was centrifuged (5 min; 300 g) and sieved through 40 μm cell strainers. Cells were cultured in REGM2 medium (Promocell) in 6 cm dishes. Confluent PTEC were exposed to γ-irradiation (γ-ray, 10 Gray) on day 6 after extraction to induce synchronized senescence. Senescence load was checked by senescence associated-beta-galactosidase (SA-ß-Gal) activity and qPCR (Cdkn2a). Cells were transferred to six-well plates on day 7. On day 14 cells were treated according to the experimental protocol and then transferred to 96-well plates on day 15. NK cells were added on day 16 and the read-out was performed on day 20.

For NK cell isolation on day 16, the NK Cell Isolation Kit Mouse was used (Miltenyi Biotec, number 130-115-818). Spleens from C57BL/6N strains B6N; 129-Ncr1tm1Oman/J and B6.129(ICR)-Tg(CAG-ECFP)CK6Nagy/J or C57BL/6-Prf1tm1Sdz/J mice were sieved through 30 μm cell strainers and labelled with the biotin-labelled antibody mix (Miltenyi Biotec). After 5 min incubation on ice, the sedimented cells (10 min, 300 g) were resuspended with buffer and anti-Biotin Micro Beads and incubated (10 min; 2°C–8°C). Cells were separated with LS-columns and resuspended with REGM2.

### Cell culture experiment

**FIGURE 1:**
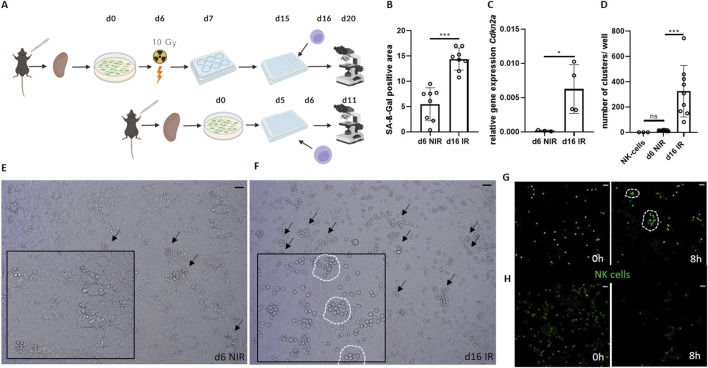
Senescent renal tubular epithelial cells increase NK cell activity *in vitro*. **(A)** Schematic of the experimental set-up of the co-culture between γ-irradiated senescent and non-senescent cells (Created by Biorender). **(B)** Quantification of SA-ß-Gal positive area (%) between senescent (d16 irradiated, IR) and non-senescent (d6 non-irradiated, NIR) cells. **(C)** Relative gene expression of Cdkn2a (p16INK4A) in primary tubular epithelial cells (PTEC). **(D)** Number of NK cell clusters per well. **(E, F)** Representative images of NK cell clusters (marked by arrows) in non-senescent (d6 NIR) and senescent (d16 IR) co-cultures. Inserts show magnifications with encircled clusters. **(G)** Senescent co-culture NK cells (GFP+) at 0 and 8 h, NK cell clusters are encircled (Supplementary Video S1). **(H)** Non-senescent co-culture NK cells (GFP+) at 0 and 8 h. Statistical significance was determined using one-way ANOVA or a non-parametric Mann-Whitney test; *p < 0.05; **p < 0.01; ***p < 0.001. Pooled data from three biological replicates in three repetitions are shown per experiment (Figure **(B)** + **(D)**). Pooled data from four biological replicates are shown per experiment. Original magnification in **(E–H)** ×400. Scale bars, 30 μm.

IL-7, and IL-15 (Sigma; Preprotech) were added to the PTEC culture at a concentration of 10 μg/mL on day 16; Cyclosporin A at a concentration of 10 mM and NKG2D-R-Antibody (BioTechne; MAB1547) at a concentration of 2 μg/mL. Respective controls were run in parallel as well as non-senescent PTEC cultures (Figure 1A). Number of PTEC was counted by DAPI staining and the senescence load was quantified by SA-ß-Gal staining. Per condition, three wells with 8 HPFs (magnification ×400) each were used for quantification. Images were analyzed with ImageJ (Schneider et al., 2012).

### Microscopy

For live cell imaging, a Zeiss 980 microscope at the Research Core Unit for Laser Microscopy of the Hannover Medical School was used. After adding NK cells to the PTEC, the co-culture was filmed for 7 h (GFP/CFP picture/3 min). Fiji was used for data transformation (Schindelin et al., 2012).

### Real-time RT-PCR

Non-irradiated and irradiated PTEC were used as seen above. NucleoSpin RNA Plus kits (Machery-Nagel) were used for isolation of RNA and a Lightcycler 480 System (Roche) was used for RNA expression quantification. Sybr green master mix was used together with primers (Eurofins genomic) for Cdkn2a (f: *CGAACTCTTTCGGTCGTACCC*; r: *CGAATCTGCACCGTAGTTGAGC*), H60b (f: *AGCCTGAGAGAGCTTTCAGAA*; r: *GGGTGTCAGAATTATGTTGGGAG*), Mult-1 (f: CAATGTCTCTGTCCTCGGAA; r: CTGAACACGTCTCAGGCACT), Mill-2 (f: TTTGGGCTGTGAGCTTCTGAG; r: AGTCCTGGTCCTGTCCTTTGT). For quantification, LightCycler 96 SW 1.1was used and the relative mRNA levels were calculated according to the 2^−ΔΔCt^ method; all samples were normalized to HPRT/actin gene expression.

### Flow cytometry

PTEC in 6 cm dishes were harvested by gentle scraping and resuspended in REGM2 medium (Promocell). Viablity was checked by microscopy and counting of viable cells (VI-Cell, Beckman). The harvested PTEC were stained with the Mult-1 antibody (rat anti-MULT1, clone 1D6, Millipore MABS1906) or H60 antibody (goat anti-H60, Thermo Fischer, PA5-47081) and then washed and stained with the corresponding secondary antibodies, anti-rat APC (Invitrogen, A10540) or anti-goat A647 (Jackson, 205-165-108). After washing with PBS/FCS, stained cells were measured using a BD FACSymphony A1 Cell Analyzer.

### Statistical analysis

Statistical significance was determined using the nonparametric Mann-Whitney test and One-way ANOVA with GraphPad Prism Software. The significance level was set to p < 0.05. No power calculation and no randomization was performed. The interpretation of results in this study primarily rests on the reproducibility of the results and the observed effect size, as shown in each figure by visualization of each individual sample (by a combination of dot plots and error bars). All methods are reported in accordance with ARRIVE guidelines.

## Results

### Senescent renal tubular epithelial cells increase NK cell activity *in vitro*

To study the interactions between NK cells and senescent tubular cells we established a murine *in vitro* model (Figure 1A). To this end, we used our standardized protocol of cellular senescence induction in PTEC, in which short-term cultured PTEC serve as non-senescent control (Liao et al., 2022; Liao et al., 2021; Berkenkamp et al., 2014). PTEC were isolated from mouse kidneys and senescence was induced by γ-irradiation followed by prolonged cell culture duration (16 days in total), while non-senescent control PTEC were only cultured for 5 days (Figure 1A). Subsequently, spleen-derived murine NK cells were added, and the renal epithelial cells were co-cultured with the NK cells for 96 h. Analysis was performed by light-microscopy, fluorescence microscopy, histochemistry or quantitative PCR (qPCR).

In this culture system, we first confirmed the induction of cellular senescence by established senescence markers SA-ß-Gal (Figure 1B) and Cdkn2a/p16INKA (Figure 1C). After senescence induction by γ-irradiation, we found that the cultured epithelial cells showed up to ∼14% SA-ß-Gal stained surface area positivity. In contrast, control cultures without irradiation showed only ∼6% SA-ß-Gal staining (∼60% reduction, p = 0.0002, Figure 1B). Accordingly, the senescence marker Cdkn2a was ∼45 times higher expressed in the irradiated epithelial cells (p = 0.0339, Figure 1C).

Next, we analyzed co-cultures of PTEC and purified mouse NK cells (Figure 1D). To quantify NK cell activity on the single cell level by microscopy, a cell-clustering analysis was performed. A cluster of NK cells was defined according to three criteria (“NK cell triad”): the cluster contains a minimum of six NK cells per cluster (1), which overlap each other, as a sign of attacking the senescent cell (2) and GFP positivity (3) as an indicator of NK cell survival.

On day 4 after NK cell co-culture, we observed numerous clusters of viable NK cells in culture wells with γ-irradiated PTEC (Figure 1D). All wells containing senescence-induced PTEC contained such clusters of NK cells (Figures 1D, F). In contrast, NK cells rarely formed clusters in the PTEC cultures without γ-irradiation (Figures 1D, E). A systematic comparison between the senescent and non-senescent co-culture from multiple independent experiments revealed significantly more clusters in the senescent group, suggesting a stronger NK cell activity (Figures 1D–F).

To support our findings by visualization with another technique, we next used live cell imaging. We visualized co-cultures of non-/senescent cells and NK cells (GFP+) for 8 hours. While there was a strong decrease in the number of NK cells in the non-senescent co-culture over time, we noticed sustained survival and a strong clustering of NK cells around senescent PTEC (Figures 1G, H and Supplementary Movie 1). This observation supports the assumption of a more aggressive attack and longer surviving NK cells in the presence of senescent target cells (Figures 1G, H). Taken together, we generated a mouse renal tubular epithelial cell culture system that allowed observing senescence-correlated clustering of NK cells.

### Clearance of senescent tubular cells by NK cells

**FIGURE 2:**
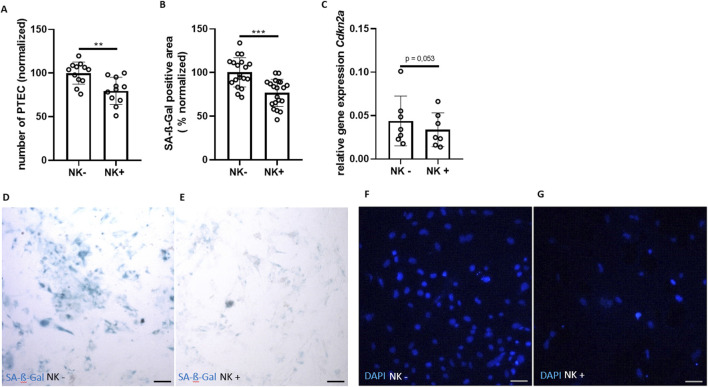
Clearance of senescent tubular cells by NK cells. **(A)** Number of PTEC (DAPI staining) cultured without NK cells (NK−) and co-cultured with NK cells (NK+) (normalized % to NK−). **(B)** SA-ß-Gal positive area (normalized to NK−). **(C)** Relative gene expression of Cdkn2a. **(D, E)** Representative pictures of SA-ß-Gal staining of PTEC without NK cells (NK−) versus co-cultured with NK cells (NK+) and **(F, G)** corresponding DAPI staining. Statistical significance was determined using a non-parametric Mann-Whitney test; *p < 0.05; **p < 0.01; ***p < 0.001. Pooled data from five biological replicates in three repetitions are shown per experiment (Figure **(A)** + **(B)**). Pooled data from six biological replicates (Figure **(C)**). Original magnification in **(D–G)** is ×400. Scale bars, 30 μm.

Having observed a clustering of NK cells in cultures of senescent PTEC, we asked whether local NK cell activity might affect the number of viable PTEC in the culture. Using the same protocol of senescence induction and NK cell co-culture from Figure 1, we compared the number of PTEC (DAPI staining) and senescence load (SA-ß-Gal staining and transcript levels of Cdkn2a) between senescent PTEC and co-culture with senescent PTEC and NK cells (Figures 2A–C).

We counted the number of viable PTEC per well and compared wells treated or non-treated with NK cells (Figure 2A). We found that addition of NK cells reduced the number of PTEC around 20% (p = 0.0036; Figures 2A, F, G). Next, we asked whether the senescent load can be reduced by NK cell treatment. As measured by SA-ß-Gal staining, NK cell treatment led to the reduction of SA-ß-Gal positivity by around 14% (p = 0.0001; Figures 2B, D, E). By qPCR, we saw a borderline significant reduction of Cdkn2a expression in PTEC after NK cell treatment (p = 0.053; Figure 2C). Taken together, the number of PTEC and the senescent load (SA-ß-Gal) were strongly reduced in the NK cell co-culture group, indicating a decrease of senescent cells through the NK cells. These findings indicate a specific identification and clearance of senescent PTEC through NK cells.

### Perforin-dependent killing of senescent renal epithelial cells

NK cells can eliminate target cells by different mechanisms (Crinier et al., 2018). To elucidate the intercellular processes between NK cells and senescent PTEC, perforin-deficient NK cells (perf ^−/−^) were used (Halle et al., 2016; Lueder et al., 2018). The same experimental set-up was used as in the experiment above, but now with co-cultures of senescent PTEC either with perforin-sufficient (perf ^+/+^) or with (perf ^−/−^) NK cells. Both groups were compared to senescent PTEC without NK cells.

**FIGURE 3:**
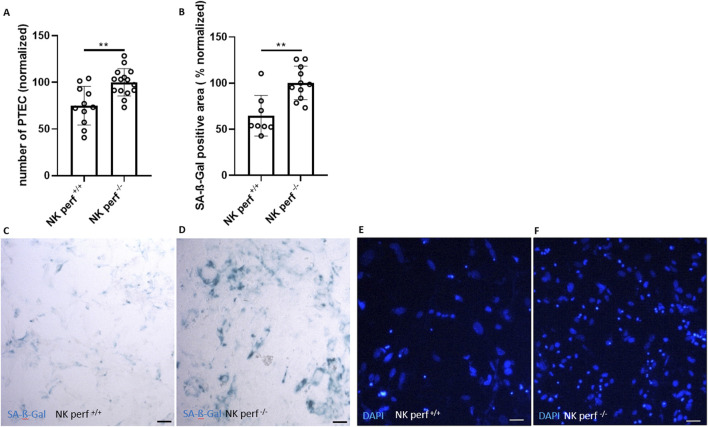
Perforin-dependent killing of senescent renal epithelial cells. **(A)** Quantification of DAPI-stained cells (number of PTEC) after co-culture with perforin-competent NK cells (perf^+/+^) and perforin-deficient NK cells (perf^−/−^) (normalized to perf^−/−^ NK cell group). **(B)** Quantification of SA-ß-Gal positive area after co-culture of PTEC with perf^+/+^ and perf^−/−^ NK cells (normalized to perf^−/−^ NK cell group). **(C, D)** Representative pictures of SA-ß-Gal staining of PTEC after co-culture with perf^+/+^ and perf^−/−^ NK cells and **(E, F)** corresponding DAPI staining for cell quantification. Statistical significance was determined using a non-parametric Mann-Whitney test; *p < 0.05; **p < 0.01. Pooled data from five biological replicates in three repetitions are shown per experiment. Original magnification in **(C–F)** is ×400. Scale bars, 30 μm.

The perf^+/+^ NK cell group showed clearance of senescent PTEC consistent with our data from Figures 1, 2. Specifically, the perf^+/+^ NK cell group showed a reduction of around 25% in PTEC number (Figures 3A, E, F compared to the perf^+/+^ NK cell group used for normalization). The perforin-expressing NK cells also triggered a 35 %-reduction in the SA-ß-Gal area (Figures 3A–F). In contrast, the perforin-deficient NK cells (perf^−/−^) did not reduce PTEC numbers or the senescence load (Figures 3A, B). Thus, the NK cell-mediated attack on senescent renal tubular epithelial cells depends on perforin-mediated killing of senescent target cells.

### NKG2D plays a crucial role in the clearance of senescent renal epithelial cells

**FIGURE 4:**
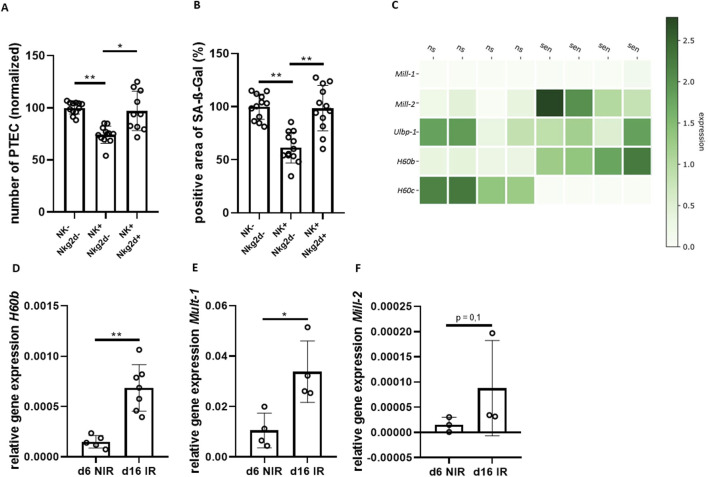
NKG2D plays a crucial role in clearance of senescent renal epithelial cells. **(A)** Quantification of DAPI-stained cells (number of PTEC) comparing senescent PTEC without NK cells (NK−), co-cultured with NK cells (NK+) and co-cultured with NK cells and NKG2D-antibody (AK) (normalized to NK− group). **(B)** Quantification of SA-ß-Gal positive area for the same groups as in **(A)**. **(C)** Heatmap of NKG2D-receptor ligands; data extracted from previously published bulk RNA-seq data of non-senescent (ns) and senescent (sen) PTEC (relative expression) (Sinning et al., 2023). **(D–F)** Quantification of transcripts of NKG2D-receptor ligands H60b, Mill-2 and Mult-1 in non-senescent (d6 NIR) and senescent PTEC (d16 IR). Significance was tested by one-way ANOVA or non-parametric Mann-Whitney test; *p < 0.05; **p < 0.01. Pooled data from four biological replicates in three repetitions are shown per experiment (Figure **(A)** + **(B)**). Pooled data from five biological replicates (Figure **(D)** + **(F)**). Pooled data from four biological replicates (Figure **(E)**).

To investigate the activating receptor on NK cells for senescent PTEC clearance, a NKG2D antibody was employed to block receptor-ligand interaction between senescent PTEC and NK cells. NKG2D has been shown to modulate immune cell activation by senescent cells (Yang et al., 2023; Deng et al., 2024). In our study, the NKG2D-blocking antibody showed an effect, resulting in a lower activity of NK cells. In comparison to the non-treated group, the antibody-treated group showed a higher number of PTEC (25%; p = 0.002) and senescence load (40%; p = 0.0001) (Figures 4A, B).

Next, we asked which signals might be responsible for the senescence-triggered NK cell activity. We analyzed changes in the transcriptome of senescent PTEC using a previously described data set (Sinning et al., 2023). We found that mRNA levels of several NKG2D-receptor ligands were upregulated in senescent PTEC, including H60b, Mult-1 (Ulbp-1) and Mill-2 (Figure 4C). Expression levels in senescent PTEC were independently tested by qPCR confirming significant upregulation of mRNA for H60b, as well as Mult-1 (Figures 4D–F). Ligand expression in PTEC was also found on the protein level using flow cytometry with Mult-1 and H60 antibodies. However, protein upregulation in senescent PTEC was minimal when compared to the non-senescent cells, and the difference did not reach statistical significance (data not shown).

### Pharmacological modulation of NK cell-mediated clearance of senescent tubular epithelial cells

**FIGURE 5:**
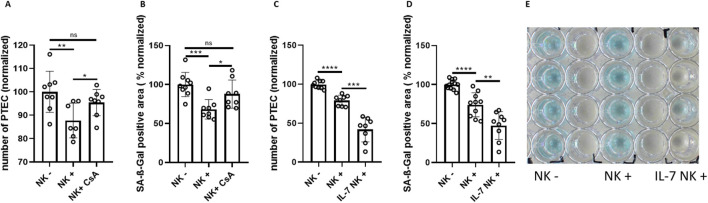
Pharmacological modulation of NK cell-mediated clearance of senescent tubular epithelial cells. **(A)** Quantification of senescent PTEC (DAPI staining) cultured without NK cells (NK−), with NK cells (NK+) and with NK cells in the presence of Cyclosporine A (CsA) (normalized to NK−). **(B)** SA-ß-Gal positive area in PTEC cultures of the same groups as in **(A)**. **(C)** Quantification of senescent PTEC (DAPI staining) cultured without NK cells (NK−), with NK cells (NK+) and with NK cells in the presence of 10 μg/mL IL-7 (normalized to NK−). **(D)** Quantification of SA-ß-Gal positive area in PTEC cultures of the same groups as in **(C)**. **(E)** Corresponding overview of SA-ß-Gal staining for experimental conditions of **(C)**. Significance was tested by one-way ANOVA or non-parametric Mann-Whitney test; *p < 0.05; **p < 0.01; ***p < 0.001, ****p < 0.0001. Pooled data from three biological replicates in three repetitions are shown per experiment.

Pharmacological immunosuppression and immunostimulatory cytokines can dramatically alter host target cells and immune cell functions. Therefore, we next wanted to test the influence of the immunosuppressive drug cyclosporine A (CsA) on NK cell-mediated clearance of renal tubular cells. In co-cultures with additional CsA, clearance of senescent PTEC by NK cells was strongly reduced. Compared to the non-treated co-culture, the number of PTEC remained significantly higher (by around 10%). Also, the SA-ß-Gal positive area after addition of CsA was larger by around 20% (Figures 5A, B). These data support the idea that CsA can downregulate the NK cell-mediated clearance of senescent renal epithelial cells.

Conversely, adding interleukin 7 (IL-7) exhibited a stimulatory effect on NK cell-mediated clearance of senescent tubular cells. While there was a reduction in PTEC number of ∼20% in the NK cell group, the addition of IL-7 was associated with a ∼50% reduction. In parallel, the SA-ß-Gal stained area was significantly smaller when IL-7 was added to the co-culture (Figures 5C–E). The strong enhancement of NK cells by IL-7 correlated with low expression of IL-7 gene in senescent PTEC (Supplementary Figure S1A). In contrast to IL-7, interleukin 15 (IL-15) showed only a trend in enhancing NK cell killing activity, while having no effect on the stained SA-ß-Gal area (Supplementary Figures S1B–D).

## Discussion

Harnessing NK cells for efficient clearance of senescent cells might provide a perspective to improve the outcome of kidney diseases and prolong kidney transplant survival (Kale et al., 2020). Our model is the first, to address direct interactions between NK cells and senescent kidney cells. We introduce a simple and robust protocol, which enables the visualization of NK cell-mediated effects on senescent renal tubular cells, as well as the quantification of NK cell activity. The model may serve as a platform for the development of novel therapies employing NK cell-based strategies for the clearance of senescent cells.

In our *in vitro* model, NK cells efficiently eliminated senescent renal epithelial cells and did not attack non-senescent cells. This observation is compatible with the notion that NK cells protect the kidney against an accumulation of cellular senescence during aging and in stress conditions. We focused purposefully on tubular cells, acknowledging that NK cell-mediated effects towards non-tubular kidney cell types (e.g., endothelial cells, interstitial fibroblasts) could be different. For example, ligands found on the surface of senescent dermal fibroblasts can inhibit NK cells via the NKG2A receptor and prevent clearance activity (Pereira et al., 2019; Wlaschek et al., 2021). While the kidney is among the most complex organs in terms of cell-type heterogeneity, tubular cells represent by far the majority of all kidney cells (Schumacher et al., 2021). Typical markers of senescence have been demonstrated consistently in aged and in stressed tubular cells across different rodent models and in human biopsies (Mylonas and Ferenbach, 2024). Thus, our primary tubular cell model provides a useful research tool for studying key mechanisms of anti-senescent effects in the kidney.

CKD is typically associated with a progressive loss of renal function, which may lead to terminal kidney failure and the need for dialysis or kidney transplantation. Kidney transplantation is the gold standard treatment option as it provides superior survival and better quality of life compared to dialysis (Kanbay et al., 2024). Although outcomes in kidney transplantation have improved over the last decades, the ongoing lack of organ availability, early graft loss due to graft rejection and side effects of immunosuppressive drugs continue to be major challenges. Undoubtedly, improved strategies to ameliorate kidney allograft health and to avoid functional deterioration have to be established. The therapeutic potential of targeting senescent tubular cells was underscored by data from zero-hour biopsies of human kidney allografts, in which the load of senescent cells correlated significantly with subsequent transplant outcomes during short- and long-term follow-up (Koppelstaetter et al., 2008; McGlynn et al., 2009; Sofue et al., 2018). Similarly, failing allografts contained strongly increased numbers of senescent cells (Melk et al., 2009; Arvizu-Hernández et al., 2010). In experimental transplantation models, cellular senescence correlated with kidney failure, whereas antagonizing cellular senescence improved the transplant outcome (Melk et al., 2005; Braun et al., 2012; Liao et al., 2022). Our co-culture model demonstrated that the calcineurin inhibitor CsA suppressed the capacity of NK cells to eliminate senescent tubular cells. This finding correlates with previous data showing reduced NK cell activity upon CsA exposure (Morteau et al., 2010; Wang et al., 2007). Correspondingly, the number of peripheral NK cells was significantly suppressed in transplant recipients treated with CsA (Vondran et al., 2012). Importantly, patients treated with belatacept, an alternative immunosuppressant, which blocks the CD80/86-CD28 co-stimulatory pathway of T-cells, maintained NK cell levels close to healthy controls (Vondran et al., 2012). Allografts of patients treated with belatacept accumulated significantly fewer senescent tubular cells over time when compared to CsA treated patients (Furuzawa-Carballeda et al., 2012). Together, these findings are compatible with the notion that CsA exposure leads to disturbed NK cell function and subsequent accumulation of cellular senescence, whereas treatment with belatacept allows healthier NK cell function and better anti-senescence surveillance. Although speculative, it seems plausible that this mechanism is a direct contributor to the demonstrated benefits and better long-term graft survival in belatacept treated transplant recipients (Divard et al., 2024; Vincenti et al., 2016).

Therapies to enable and improve immune cell function often involve immune stimulatory factors. We found that IL-7 significantly enhanced the senolytic efficiency of NK cells in our culture system, while IL-15 had only minor effects. However, broad enhancement of immune cell activity by cytokines might have unspecific and difficult-to-control effects. In the renal field, this could be particularly challenging, because the immune response is often overactive, e.g., autoimmune disease or allogeneic kidney transplantation. As an alternative, it has been suggested to implement specific antigen-directed therapies, which allow clearance of senescent cells with high selectivity. Promising results in eliminating senescent cells have been obtained with chimeric antigen receptor (CAR) T-cells (Deng et al., 2024; Yang et al., 2023; Amor et al., 2024; Amor et al., 2020). In the first study using this strategy, CAR T-cells were developed to target the protein urokinase-type plasminogen activator receptor (uPAR) (Amor et al., 2020). Although this approach achieved an efficient clearance of senescent cells, it is questionable whether it is applicable to the diseased kidney, where uPAR is broadly expressed in different cell types during cell stress and does not seem to correlate well with cellular senescence (Zhang and Eddy, 2008). An alternative strategy employs NKG2D-CAR T-cells, which exploit the increased expression of NKG2D ligands on the surface of senescent cells (Deng et al., 2024). NKG2D ligands can mediate activating signals to attacking NK cells, as has been described in the context of immune cell-mediated attack on virus-infected cells (Wensveen et al., 2018). Therefore, we were interested in the impact of NKG2D signals on NK cell-mediated attack on senescent renal tubular epithelial cells. In fact, we found NKG2D receptor ligands, particularly H60b and Mult-1, to be significantly upregulated on the mRNA level in our data for senescent cells and blocking of the NKG2D receptor blunted the activity of NK cells towards senescent cells. Fittingly, it was shown that significant reductions of senescent cells achieved by NKG2D-CAR T cells were paralleled by a decrease in Mult-1 and H60b expression in various tissues including the kidney (Yang et al., 2023). With a focus on Mult-1 and H60b as potential ligands, we found a senescence-associated upregulation of mRNA levels in PTEC, but as this relative upregulation in senescent compared non non-senescent cells was not found on the protein level, additional ligands are likely to be involved.

The present study has some limitations. The first limitation is the fact that we restricted our research to the interaction of only two specific cell types (i.e., NK cells and tubular cells), which does not allow to capture the inherent complexity of the multi-cell type situation in the kidney. Mechanisms of NK cell interaction with other kidney cell types, such as endothelial cells and fibroblasts have not been addressed in our study. Together with other modifying factors of the internal milieu (e.g., cytokines) it is likely that the presence of other neighbouring cell types could have impacted our results. This should be studied in future studies. Another limitation of our model is that we here have focused on mouse cells. Given the discrepancies in ligands between human and murine cells (Liu et al., 2019), future experiments will have to include human tubular epithelial cells and NK cells. Additionally, future studies will have to validate the findings in animal experiments *in vivo* and should attempt to include clinical sample validation.

In summary, we introduce a novel easy-to-use cell culture model, which may serve as a platform to study NK cell-based strategies for the clearance of senescent kidney cells. Our data show the pivotal involvement of NKG2D receptor signaling with a potential involvement of ligands like H60b and Mult-1 in the NK cell-mediated elimination of senescent renal tubular cells. Understanding the mechanisms of immunosurveillance by which senescent cells are controlled and eliminated by the immune system presents a promising way for the development of therapeutic interventions to mitigate age-related or senescence-associated kidney disorders.

## Data Availability

The raw data supporting the conclusion of this article will be made available by the authors, without undue reservation.

## Supplementary material

SUPPLEMENTARY FIGURE S1

**(A, B)** Quantification of transcripts of interleukin 7 (IL-7) and interleukin 15 (IL-15) comparing non-senescent (d6 NIR) and senescent PTEC (d16 IR). **(C)** Quantification of PTEC (DAPI staining) comparing senescent PTEC after culture with NK cells (NK+) and with additional IL-15 (normalized to NK−). **(D)** SA-ß-Gal positive area for PTEC cultures corresponding to experimental conditions of **(C)** (normalized to NK−). Significance was tested by one-way ANOVA or non-parametric Mann-Whitney test; *p < 0.05; **p < 0.01; ns, non-significant. Pooled data from at three independent experiments are shown, with a minimum of five biological replicates.

SUPPLEMENTARY VIDEO S1

**(A)** Fluorescence microscopy video 1: NK cells (GFP+) and non-senescent PTEC (RFP+) for 8 h. **(B)** Fluorescence microscopy video 2: NK cells (GFP+) and senescent PTEC (RFP+) for 8 h.
