# Supplementary figures and images for: Mechanisms of natural killer cell-mediated clearance of senescent renal tubular epithelial cells
Source: Front Cell Dev Biol. 2025 Jun 30;13:1597230. doi: 10.3389/fcell.2025.1597230 (PMC12256444; doi:10.3389/fcell.2025.1597230)

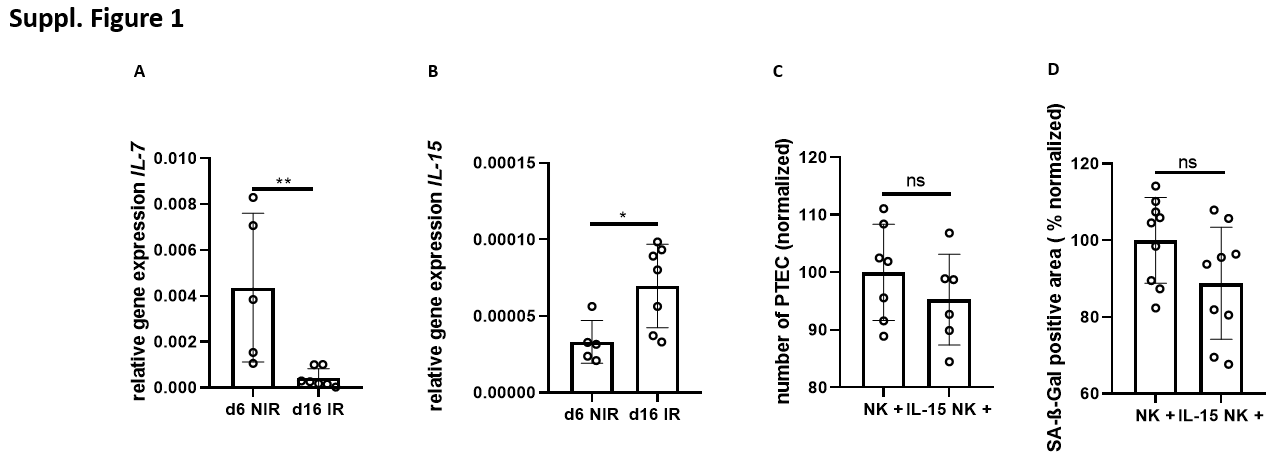

Supplement: Supplementary file 2 [file Image1.tif]
